# Simulation and Design of Circular Scanning Airborne Geiger Mode Lidar for High-Resolution Topographic Mapping

**DOI:** 10.3390/s22103656

**Published:** 2022-05-11

**Authors:** Fanghua Liu, Yan He, Weibiao Chen, Yuan Luo, Jiayong Yu, Yongqiang Chen, Chongmiao Jiao, Meizhong Liu

**Affiliations:** 1 Key Laboratory of Space Laser Communication and Detection Technology, Shanghai Institute of Optics and Fine Mechanics, Chinese Academy of Sciences, Shanghai 201800, China; fhliu@siom.ac.cn (F.L.); wbchen@siom.ac.cn (W.C.); leekp@mail.ustc.edu.cn (Y.L.); yongqiangchen@siom.ac.cn (Y.C.); cmjiao@siom.ac.cn (C.J.); meizhongliu@siom.ac.cn (M.L.); 2 Center of Materials Science and Optoelectronics Engineering, University of Chinese Academy of Sciences, Beijing 100049, China; 3 Department of Guanlan Ocean Science Satellites, Pilot National Laboratory for Marine Science and Technology, Qingdao 266237, China; 4 School of Civil Engineering, Anhui Jianzhu University, Hefei 230601, China; yujiayong@ahjzu.edu.cn; 5 School of Physics and Optoelectronic Engineering, Hangzhou Institute for Advanced Study, University of Chinese Academy of Sciences, Hangzhou 310024, China

**Keywords:** lidar, remote sensing, Geiger-mode lidar, simulation model, topographic mapping, circular scanning, point cloud generation, data compression

## Abstract

Over the last two decades, Geiger-mode lidar (GML) systems have been developing rapidly in defense and commercial applications, demonstrating high point density and great collection efficiency. We presented a circular scanning GML system simulation model for performance prediction and developed a GML system for civilian mapping. The lidar system used an eye-safe fiber laser at 1545 nm coupled with a 64 × 64 pixels photon-counting detector array. A real-time data compression algorithm was implanted to reduce half of the data transmission rate and storage space compared to the uncompressing situation. The GML system can operate at aircraft above-ground levels (AGLs) between 0.35 km and 3 km, and at speeds in excess of 220 km/h. The initial flight tests indicate that the GML system can operate day and night with an area coverage of 366 km^2^/h. The standard deviations of the relative altimetric accuracy and the relative planimetric accuracy are 0.131 m and 0.152 m, respectively. The findings presented in this article guide the implementation of designing an airborne GML system and the data compression method.

## 1. Introduction

Airborne laser scanning (ALS) is a widely used active remote sensing technique for recording the surface and terrain of the earth, which generates accurate digital elevation models and serves in some basic applications, such as civilian surveying and mapping, biomass measurement, bathymetry, and military surveillance [1,2,3,4,5]. Commonly, ALS mapping lidars include digitized waveform lidars, single-photon lidar (SPL), and Geiger-mode lidar (GML) [6]. For the digitized waveform lidars (or linear mode lidars), the received photons are converted into an electric signal proportional to the incident laser intensity by a detector working in linear mode. The target distance and the reflect intensity are contained in the echo signal, which can be used to generate a three-dimensional (3D) point cloud and to provide target reflectance or material properties via radiometric calibration. In contrast to linear mode lidar (LML), which typically requires hundreds of detected photons, single-photon and Geiger-mode lidar can achieve range measurements by just a few returning photons per pulse, due to the detector’s sensitivity to individual photons. For the single-photon lidar, a typical system is the multibeam Single Photon LiDAR (SPL) developed by the Sigma Space Corporation, which uses a photomultiplier tube with a very short dead time [7]. For the Geiger-mode lidar, a typical lidar is the Harris IntelliEarth™ Geospatial Solutions Geiger-mode LiDAR sensor, which utilizes a large-pixel-format focal plane array (FPA) detector [8].

Comparisons between LML, SPL, and GML were demonstrated in references [9,10,11,12,13,14]. The results showed that the measurement precision of SPL and GML is lower than that of LML on rough surfaces, and the measurement precision of these systems is the same on smooth surfaces [15]. SPL and GML have higher area collection efficiency than LML, due to the high sensitivity of the detector array [16]. In addition, the ultra-high point density image generated by SPL and GML can improve foliage penetration to better sample bare earth and reveal the infrastructure details [8]. In recent decades, the Lincoln Laboratory developed a series of lidar systems based on arrays of Geiger-mode avalanche photodiode (GMAPD) detectors [17,18,19]. These systems validate the excellent utility of GML in foliage penetrating imaging and defense operations mapping. Recently, the characteristics of a high area coverage rate and high point density open up new applications for GML in humanitarian aid and disaster relief [20].

A proper simulation model of airborne GML is essential for the prediction of lidar performance and the optimization of lidar design. The performance of GML in point density and ranging accuracy is significant for the quality of the point cloud product. The authors in [21] proposed a simulation of a GML system to assess system performance and generate sample data. However, the simulation has not yet been experimentally verified. In addition, the GML system datasets are much larger than those collected by LML systems. For a 256 × 64 pixels GMAPD array capable of readout rates in excess of 8 kHz, the raw data recorder rate is about 250 MB/s [18]. With the increasing pixel format of the GMAPD array, the data recorder rate will be extremely high in the future. The high recorder rate is challenging for real-time 3D imaging, and huge storage space is required for data curation. Therefore, the establishment of a data compression algorithm and the airborne verification experiment of the simulation model are the keys to improving the performance of the GML system. However, these aspects have not yet been fully studied.

In this paper, we present a simulation model of circular scanning airborne Geiger-mode FPA imaging lidar. The point density and the optimal rotational speed of the scanner can be predicted, which is significant in designing an airborne GML system. The circular scanning airborne GML system we developed can operate at AGLs between 0.35 km and 3 km. The lidar system employed a 64 × 64 pixels InGaAs/InP detector array to obtain the ranging profiles of surfaces. In addition, we developed a real-time data compression algorithm that is realized by only storing a small range of data containing the target in the range gate. This real-time data compression algorithm was applied in lidar to reduce half of the data transmission rate and storage space compared to the uncompressing situation. We organized several initial flight tests that were conducted in Wuhu City and Qionghai City to validate the simulation model and the real-time data compression method.

This paper aims initially, in Section 2, to briefly introduce the principle of circular airborne Geiger-mode lidar (GML). Then, we present an overview of our circular scanning airborne GML system in Section 3. Section 3 provides the details of the real-time data compression method and the point cloud generation method. Section 4 presents the airborne experimental results and the performance assessments. Finally, Section 5 summarizes the main findings and provides an outlook for future research.

## 2. Principle of Airborne Geiger-Mode FPA Imaging Lidar

**Figure 1:**
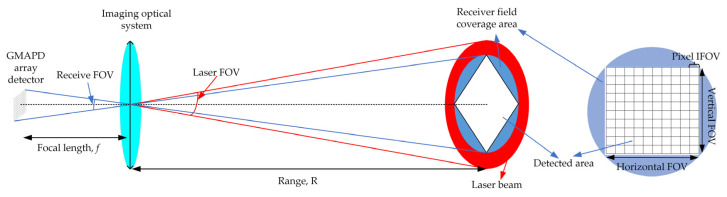
Schematic diagram illustrating the geometry of the Geiger-mode FPA lidar.

**Figure 2:**
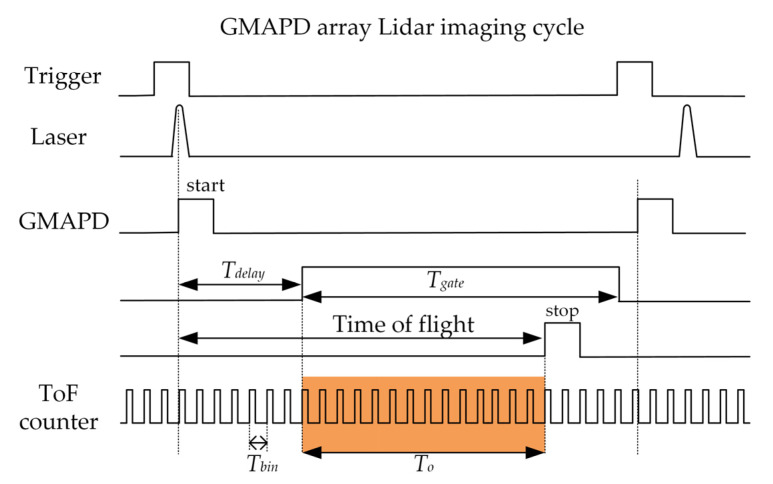
The timing sequence of a Geiger mode lidar imaging cycle.

Figure 1 shows the basic principle of a Geiger-mode FPA Lidar. The scenery is illuminated by a laser pulse. Each pixel of the Geiger-mode FPA detector precisely measures the time of flight (ToF) of the photons reflected off the surface within the instantaneous field of view (IFOV) to determine the three-dimensional coordinate. Figure 2 illustrates the timing sequence of a Geiger mode lidar imaging cycle. *T_bin_* is the timing resolution of the ToF counter integrated with the GMAPD. Lidar triggers the laser to emit a laser pulse and, in the meantime, triggers the ToF counter to start timing. The range gate delay time (*T_delay_*) is set based on a priori knowledge of the approximate distance to the target. Following the range gate delay, the range gate is open and the range gate duration (*T_gate_*) is chosen to completely encompass the volume of interest. During the range gate duration, each GMAPD in the detector array is operated with a reverse-bias voltage above the avalanche breakdown voltage to detect the reflected photons. Once a pixel detects the photons, the ToF counter integrated with the pixel will stop timing. Finally, when the range gate duration is over, the over-bias voltage will be removed, the array TOF values will be read out, and the array is then ready for the next cycle.

For the airborne Geiger-mode FPA imaging lidar, assume that the terrain within the receiver field of view (FOV) is illuminated by the laser pulse and is imaged onto a solid-state *N* × *N* pixels FPA detector. The mean number of signal photoelectrons per laser fire detected by the receiver can be calculated by the lidar range, as shown in Equation (1) [22]:

(1) $$n_{r}=\eta_{atm}^{2}\eta_{t}\eta_{r}\eta_{d}\rho \cos\sigma \frac{E_{t}\lambda }{hc}\frac{A_{r}}{\pi R^{2}}$$

where $\eta_{atm}$ is the one-way atmospheric transmission, $\eta_{t}$ and $\eta_{r}$ are the transmission efficiency of the transmitter and the receiver optics, respectively, $\eta_{d}$ is the detector average photon detection efficiency (PDE), ρ is the target reflectivity, σ is the target slope within the laser footprint, *E_t_* is the laser pulse energy, λ is the laser wavelength, *h* is the Planck’s constant, *c* is the velocity of light in vacuum, *A_r_* is the collecting area of the receiving telescope, and *R* is the distance from lidar to surface.

The one-way atmospheric transmission is determined by Equation (2) [23]:

(2) $$\eta_{atm}=\exp(-kR)$$

where the scattering coefficient *k* can be expressed according to visibility and wavelength by the following expression:

(3) $$k=\frac{3.91}{V}{\left(\frac{\lambda }{550}\right)}^{-q}$$

where *V* is the visibility in km and λ is the wavelength in nm.

The coefficient *q* can be calculated by:

(4) $$q=\{\begin{array}{c} 1.6\\ 1.3\\ 0.585V^{1/3} \end{array}\begin{array}{c} V\geq 50\;\mathrm{km}\\ 6\;\mathrm{km}\leq V<50\;\mathrm{km}\\ V<6\;\mathrm{km} \end{array}$$

To calculate the mean number of signal photons returned to each GMAPD in the FPA camera, several system configurations should be considered, including: (1) the far-field intensity distribution of the transmit laser beam; (2) the ratio of the detected area to the receiver field coverage area; and (3) the effective detector fill factor of the GMAPD elements. Generally, the transmit laser beam can be shaped as a Gaussian beam, a super-Gaussian beam, or a flat-top beam to illuminate the surface. Recently, Kim et al. provided a detailed description of a GML system that used an unshaped Gaussian beam as the transmitter [21]. In addition, the ASOTB employed a pair of commercial off-the-shelf cylindrical lenses to shape the circular laser beam for matching the GMAPD receiver’s 4-to-1 aspect ratio [19]. For the most available GMAPD elements, micro lenses are used to ensure that every returned photon incident on the array efficiently couples to the detector. The effective detector fill factor can be up to above 60%.

Here, to simplify the model, we assumed that the intensity distribution of the laser footprint is uniform and that the transmitter FOV is approximately the same as the receiver FOV. Therefore, for an *N* × *N* pixels GMAPD array, the mean number of photons returned to each pixel can be calculated by Equation (5):

(5) $$n_{s}=\frac{\delta_{r}\delta_{f}}{N*N}n_{r}$$

where $\delta_{r}$ is the ratio of detected area to receiver field coverage area and $\delta_{f}$ is the effective detector fill factor of the Geiger-mode FPA detector.

The noise photons generated in the single-photon detector mainly come from the solar noise background and the dark count, which will pollute the 3D point cloud. For each GMAPD in the array, the received mean number of background noise photons within a range gate duration is described by Equation (6):

(6) $$n_{b}=\rho A_{r}\eta_{atm}\eta_{r}\eta_{d}\Delta \lambda T_{gate}E_{\lambda }\frac{\lambda }{hc}\frac{\theta_{r}{}^{2}}{4}\cos\theta_{sun}$$

where Δλ is the bandwidth of the optical filter, $\theta_{r}$ is each individual pixel’s IFOV, $\theta_{sun}$ is the subtended angle between the sun and the surface normal, and $E_{\lambda }$ is the wavelength-dependent solar spectral illuminance on the surface. For the wavelength of 1545 nm, the value of $E_{\lambda }$ is about 0.27 W/(m^2^·nm).

The PDE and the dark count rate (DCR) of every pixel in the GMAPD camera are dependent on the integrated circuit technology, the reverse-bias voltage, and the thermal distribution in the array, but most of the pixels have almost the same PDE and DCR [24]. Hence, the total noise photoelectrons detected by the individual GMAPD can be determined by Equation (7):

(7) $$n_{n}=n_{b}+n_{d}=n_{b}+{\bar{f}}_{d}*T_{gate}$$

where $n_{d}$ is the number of noise photoelectrons caused by the dark count in every pixel and ${\bar{f}}_{d}$ is the FPA detector average DCR in Hz.

As the echo signal is weak, the number of detected photoelectrons follows Poisson statistics [21,22]. The distribution is determined by Equation (8):

(8) $$P(m)=\frac{\exp(-n)n^{m}}{m!}$$

where *n* is the photons in the *T_bin_* and *P(m)* is the probability that *m* photoelectrons are detected during the imaging cycle.

Normally, the dead time of GMAPD is 50 nanoseconds when the single-photon avalanche diode is actively quenched. The lidar can detect the surface many times during the range gate. However, the GMAPD array detector implemented in our lidar system has only one measurement opportunity during the whole range gate, due to the limit of the readout integrated circuit. Referring to the timing sequence in Figure 2, *T_o_* is the time delay before the target signal is detected. Then, the detection probability of zero noise photons being detected before the target is determined by Equation (9):

(9) $$P_{non}=P(m=0)=\exp(-\frac{T_{o}}{T_{gate}}n_{n})$$

Therefore, the detection probability of the photons signal photons is determined by Equation (10):

(10) $$P_{s}=P_{non}(1-P(m=0))=\exp(-\frac{T_{o}}{T_{gate}}n_{n})(1-\exp(-n_{s}))$$

As was meticulously described in [1], to map unobstructed solid surfaces in the daylight, there are only three possible outcomes for a given GMAPD pixel-per-imaging cycle: (1) a surface photon is detected; (2) no photons are detected; or (3) a noise count is detected. In addition, the probability of these three situations was strictly described by Degnan [1] when the range gate is approximately centered on the surface. For the range gate duration of 4096 ns, we usually set an appropriate range gate delay time to make sure that three-quarters of the range gate duration is approximately above the local ground altitude. Therefore, the probability of these three situations is determined by the following equations:

(11) $$P_{s}=\exp(-\frac{3}{4}n_{n})[1-\exp(-n_{s})]$$

(12) $$P_{z}=\exp(-n_{n})\exp(-n_{s})$$

(13) $$P_{n}=1-P_{s}-P_{z}$$

where $P_{s}$ is the probability that a surface photon is detected, $P_{z}$ is the probability that zero photons are detected, and $P_{n}$ is the probability of detecting a noise count.

**Figure 3:**
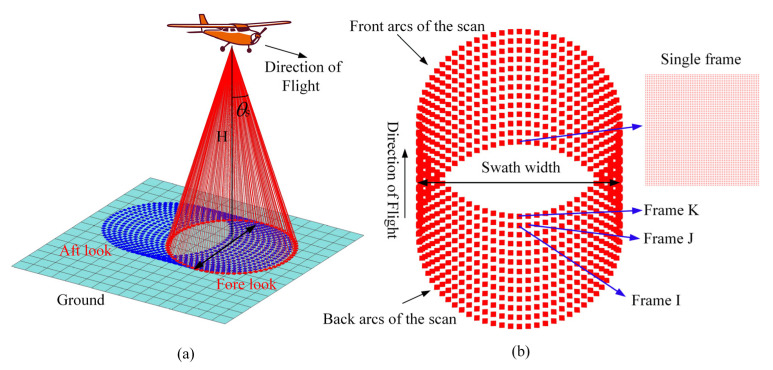
(**a**) Diagram showing the circular ground scanning pattern and coverage of an airborne Geiger-mode FPA imaging lidar; (**b**) simulation of lidar point distribution on the ground.

For a circular scanning airborne Geiger-mode FPA imaging lidar, the scanner covers the full swath width at least twice the per flight line with a fore and aft look, as shown in Figure 3 [25]. The back and front arcs of the scan are treated as individual swaths during point cloud generation. The single frame data are the ranging data acquired by the GMAPD array detector during one imaging cycle. As simulated in [7], for the circular scanning pattern, although density increases significantly at the lateral edge of a strip, the density for much of the swath width is homogenous. Hence, the average point density can represent the point density of the majority scanning area. It can be predicted by dividing the total number of detected points by the total surface area scanned during the same time interval, i.e.,

(14) $$\bar{P}=\frac{N*N*P_{s}*f_{rep}}{2H*\tan(\theta_{s})*v}$$

where $f_{rep}$ is pulse repetition frequency, *H* is flying height, *v* is flying speed, and $\theta_{s}$ is the conical scan angle to nadir. In Equation (14), the numerator and the denominator are the number of detected ground points and the swept area in one second, respectively.

An appropriate rotational speed must be set at a different flying altitude to reduce the scanning voids. To simplify the model, we assumed that the lidar optical system is ideal. Thus, the imaging area of the *N* × *N* APD array on the ground is quadrate and the side length can be determined by Equation (15):

(15) $$L=\frac{2H}{\cos(\theta_{s})}*\tan(\frac{N*\theta_{r}}{2})$$

**Figure 4:**
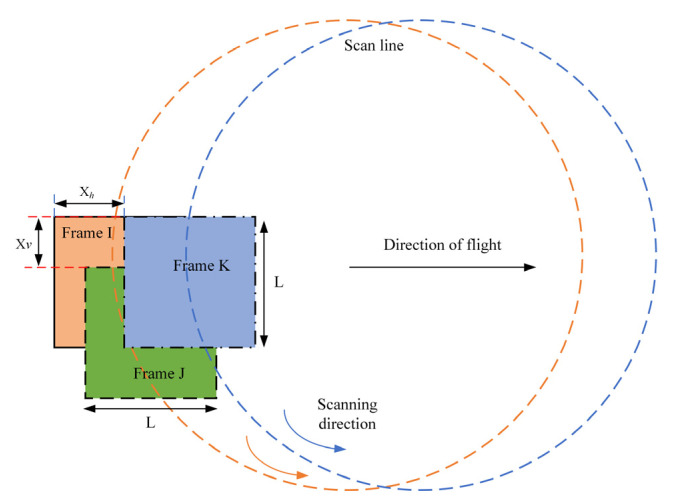
Illustration showing the scan path on the ground of the airborne GML employing a circular scanner.

For the circular scanner, the back and front arcs of the scan can be treated as individual swaths. For the backward swath, as shown in Figure 3 and Figure 4, frame I and frame J are adjacent along the scan line in the center of the swath, while frame I and frame K are adjacent across the scan line. *X_h_* is the horizontal space length between frame I and frame K and *X_v_* is the vertical space length between frame I and frame J. To avoid the scanning voids in the backward swath, when the *X_v_* value is as large as *L*, we can obtain the maximum number of rotations of the scanner per second by Equation (16)

(16) $$r_{\max}=\frac{f_{rep}*L}{2\pi H*\tan(\theta_{s})}$$

When the *X_h_* value is equivalent to *L*, we can obtain the minimum number of rotations of the scanner per second by Equation (17)

(17) $$r_{\min}=\frac{v}{L}$$

Therefore, when the values of *X_h_* and *X_v_* are equivalent, the mapping area is evenly covered. The optimal rotational speed of the scanner is determined by Equation (18):

(18) $$r_{opt}=\sqrt{\frac{v*f_{rep}}{2\pi H*\tan(\theta_{s})}}$$

Considering that the forward swath covered the same area, the rotational speed range of the scanner can be larger than the range calculated by Equations (13) and (14), but the optimal rotational speed is still the same.

## 3. System Overview

**Figure 5:**
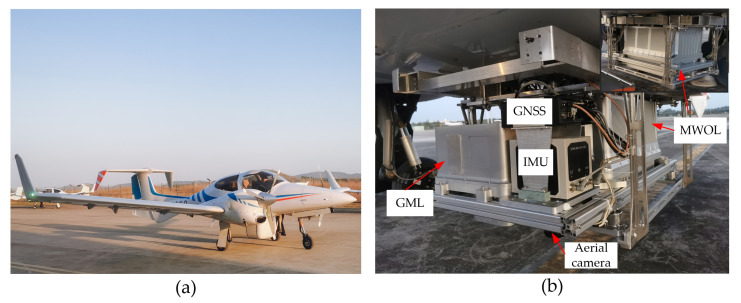
(**a**) The Diamond DA-42MPP aircraft on the tarmac at Wuhu Xuanzhou Airport; (**b**) photograph of the airborne multispectral lidar system mounted on the aero belly. A back view of the system is displayed in the inset.

The airborne GML is one of the important components of our multispectral lidar system for civilian surveying and mapping, biomass measurement, and bathymetry. As shown in Figure 5a, the multispectral lidar system was fitted to the belly pod of the twin-engined Diamond DA42 multipurpose platform aircraft. This relatively low operating-cost platform offered flight endurance in excess of 3 h at speeds of 200–365 km/h and electrical power for research activities.

This multispectral lidar system consists of GML, multi-wavelength ocean lidar (MWOL), a high-resolution aerial camera, and an integrated navigation system (INS), as shown in Figure 5b. The INS includes a global navigation satellite system (GNSS) and an inertial measurement unit (IMU). The high accuracy position and the attitude of the multispectral lidar are provided by the INS. It also offers triggering via a pulse generator with a pulse-per-second (PPS) signal, which synchronizes the MWOL and GML sensors. The MWOL was designed to work in linear mode and is mainly applied to intertidal zone surveying and bathymetric mapping. The MWOL’s solid-state laser was developed to generate pulse lasers with wavelengths of 1064 nm, 532 nm, 486 nm, and 355 nm. The target intensity of different wavelengths can be obtained for point cloud classification and other applications. In addition, a palmer scanner was utilized in MWOL with a scanning FOV of 30°, and the nominal AGL range was 0.3 km to 0.5 km. As a combination, the GML operating at a wavelength of 1545 nm was designed to acquire a high-density lidar image to compensate for the MWOL’s low-density point cloud.

**Figure 6:**
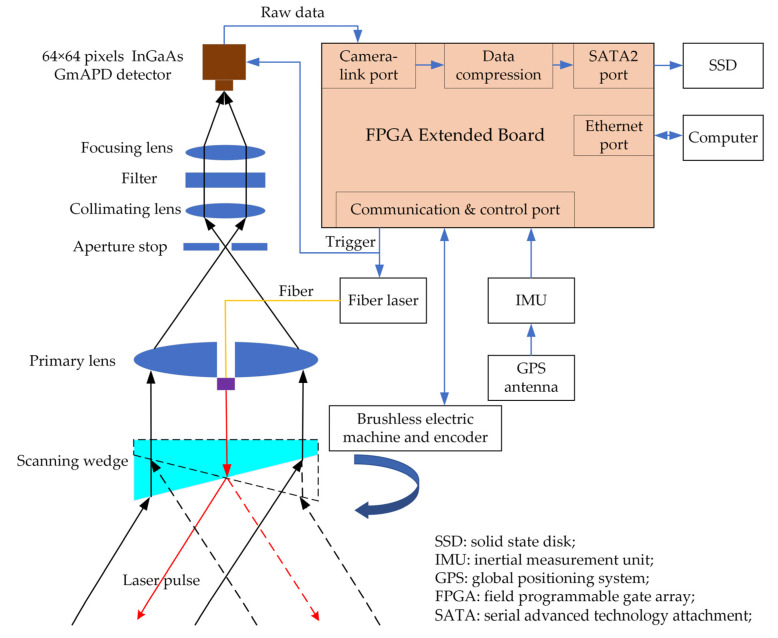
Block diagram of the circular scanning airborne Geiger-mode FPA imaging lidar. FPGA: field programmable gate array; SATA: serial advanced technology attachment; TB: terabyte; SSD: solid-state disk.

A block diagram of the detailed components of our GML system is presented in Figure 6. A host computer sends control instructions to the GML system through a user datagram protocol and displays the raw ranging data in rea time. The primary technical specifications of the GML system are listed in Table 1. Furthermore, the details of the system, the simulation results of the proposed system, and the method of point cloud generation are described in the following sections.

### 3.1. Laser and Detector Modules

The electro-optical receiver is a 64 × 64-pixel, 50-micron-pitch, photon-counting GMAPD array. Normally, the Geiger mode APD detector operates at a reverse-bias voltage above the avalanche breakdown voltage. It generates large currents that can be detected by digital timing circuitry when just a few photons hit the APD sensing area. The mean photon detection efficiency of the APD reaches 20%. The dark count rate of each APD is about 5 kHz, achieved by cooling the APD array to −20 ℃ during normal airborne operations. The solar noise per pixel is minimized through a narrow bandpass spectral filter, a small receive telescope 7.5 cm in diameter, and a small receive IFOV.

The GMAPD receiver array is sensitive to single photons, thereby relaxing the requirements of the transmitted laser power. A low-power pulsed fiber laser could be chosen to replace the high-power solid-state laser. Therefore, the requirements of overall system power, weight, and size would be reduced, which is important for airborne lidar applications. In this GML system, a diminutive, pigtailed, pulsed fiber laser source with an operating wavelength of 1545 nm was employed. The pulse width of the fiber laser was approximately 700 ps. The fiber laser emitted a Gaussian beam with a pulse repetition rate of 20 kHz and average power of 260 mW.

### 3.2. Optical System Design and Scanner Module

In the transceiver unit, the laser pulse is directed to the ground by a beam expander and a scanner. The returning light is collected by a homemade primary lens and collimated by a collimating lens. Then, the light moves through a 25.4 cm diameter, 3 nm wide narrow-band optical filter to reduce the amount of background, which affects the detector during daytime operations. Finally, the returning light is focused on the GMAPD array by a lens. In order to ensure that the laser path is coaxial with the optical axis of the main telescope, a 15 cm diameter hole was bored in the middle of the primary lens, as shown in Figure 6. Moreover, the mounting space of the transmitting optics was reduced, which is of great benefit for space-starved airborne lidar operations. A further advantage is that the coaxial optical system is more convenient to adjust and align than the off-axis optical system.

The IFOV of each pixel in the detector array was approximately 60 μrad. The far-field divergence of the transmitted beam was fixed to 5.5 mrad after the laser passed through the transmitted optics, which is larger than the received FOV and offers a high coaxial tolerance. The GML can be regarded as a range camera. Hence, every single-frame data can be seen as a picture with distance. As is the case with conventional imaging systems, aberrations such as spherical, coma, astigmatism, and field curvature caused by the GML receiver optics cannot be ignored. In this GML, all the lenses were chosen as aspherical lenses to minimize the influence of aberrations. The focal length of the imaging optical system was 833.33 mm. The diameter of the circle of confusion was optimized to 45 μm, which was within the pixel size.

The scanner is a scanning wedge driven by a high-performance brushless motor. The rotational speed of the motor was more than 2000 revolutions per minute (RPM). However, the actual laser location on the surface is significant for the point cloud generation. Therefore, the installation angle and slope of the wedge prism should be measured with high accuracy. The rolling axis of the scanner was set colinear to the optical axis of transceiver optics. As shown in Figure 6, the upper side of the wedge prism was perpendicular to the transceiver optical axis, while the slopy side was toward the ground to reduce the number of refractions in the prism. The wedge angle of the prism was 19.536°, which was measured by a bridge coordinate measuring machine. Then, the scanning angle could be accurately calculated based on the refractive equation.

### 3.3. Data Sampling and Real-Time Data Compression

The FPGA triggers the laser to emit a pulse. Then, the timers behind each GMAPD pixel are triggered to count after a delayed time, depending on flying altitude. These timers adopt 12-bit counters to record the time of flight (ToF) of echo events with the timing resolution of 1 ns. Therefore, the data recorder/acquisition server needs 2 bytes to store the ToF for each pixel. The storage rate will reach at least 156 MB/s with a frame rate of 20 kHz. However, the storage rate may be challenging for the SATA2 interface, and a huge storage space is required for data curation. Therefore, developing real-time data compression is necessary to reduce the storage rate.

For the GML system with a small FOV, we assumed that the number of targets in a single frame is only one. Then, the reflected photons of the target could be detected by most pixels in the array, and the ToF of these pixels was approximately the same in this frame. We recoded a small distance range that contained the primary target by 8 bits of memory space, while the other ToF was discarded. In this case, the 12-bit ToF was compressed to 8 bits. The recorder server needed only one byte to store the ToF for each pixel, which meant that the data storage rate could be reduced to 78 MB/s. Compared to the uncompressing situation, the data storage space was reduced by half. The real-time compression algorithm could be easily realized in FPGA by the following steps:

Step 1: For single-frame ranging data acquired by the GMAPD, the peak count was found in the time-correlated histogram as the reference value A.

Step 2: Using each pixel’s ToF value (B1, B2, …, B4096) minus A, the difference value (C1, C2, …, C4096) was obtained.

Step 3: If the difference value was located in [−63, 64], we used symbol one to tag this pixel’s ToF as valid and used 7 bits to record the difference value calculated by Step 2. In addition, if the difference value was beyond [−63, 64], we used the symbol zero to tag this pixel’s ToF as invalid.

**Figure 7:**
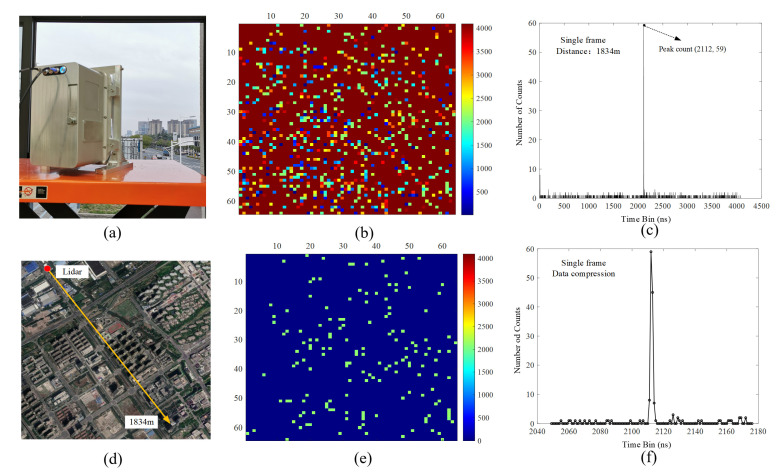
(**a**) The scenario of the lidar is imaging a building; (**b**) picture of the raw counting value for each pixel in a single frame and the coloring is set according to the count; (**c**) picture of the time-correlated histogram before data compression; (**d**) the distance between lidar and building is 1834 m shown in Google Earth; (**e**) picture of the counting value for each pixel in a single frame after data compression; (**f**) picture of the time-correlated histogram after data compression.

We created a scenario where the lidar images a building at a fixed distance of 1.834 km. As shown in Figure 7, the pixel’s ToF value near the target was reserved, while the others were abandoned after the real-time data compression algorithm. For single-frame data, the root mean square (RMS) range precision of the building was 0.09 m after noise filter processing.

**Figure 8:**
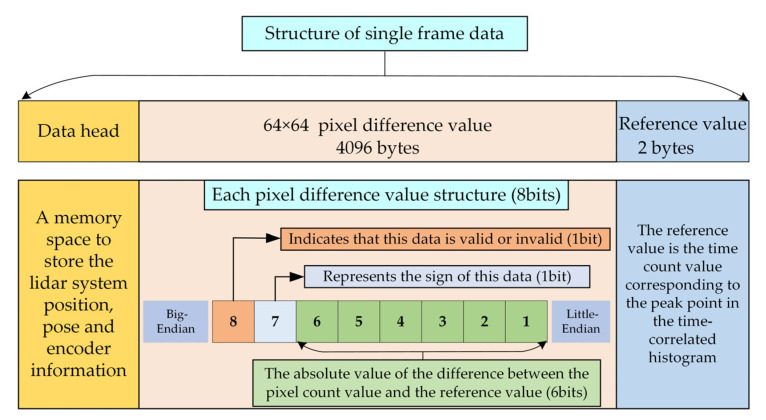
Block diagram of the data structure stored in SSD.

Moreover, in order to match the timestamps between the scanning system, the detector system, and the INS system, the GPS’s PPS signal was used as the synchronization pulse and the internal oscillator clocks on the FPGA for further precision. Finally, the synchronized information, including lidar system position, pose, encoder, and the compressed counting values, was saved in the SSD. The stored structure of data is shown in Figure 8.

### 3.4. System Analysis and Simulation Results

The requirements of laser power for a GML system depend on several parameters, which were analyzed in Section 2. For our GML lidar, the major technical specifications are shown in Table 1. We assumed that the plane’s flying speed, the target reflectivity, the two-way atmospheric transmission, and the visibility were 220 km/h, 20%, 81%, and 15 km, respectively. By substituting these parameters into the simulation model, the simulation results (shown in Table 2) included the mean number of detected signal photons per pixel per pulse, the mean number of noise photons during range gate per detection cycle, the point density, and the scanner rotational speed.

### 3.5. Point Cloud Generation

For an airborne lidar, a series of coordinate frame transformations are executed to calculate the geo-referencing of points. First, the 3D coordinates of the laser footprint in the lidar reference frame (LRF) were obtained based on the lidar geometric model. Then, the points in the LRF were transformed to the INS reference frame (IRF) according to the boresight angles. After that, the points in IRF were transformed to the local geodetic frame (LGF) through three attitude angles (roll, pitch, and yaw) provided by the IMU. Finally, the coordinates of the points in LGF were transformed to ECEF reference frame (ERF). The parameters of the transformation from LGF to ERF (including the latitude, the longitude, and the ellipsoidal height) were provided by the GNSS. Referring to the literature [26,27,28,29], the generic georeferencing of a typical lidar survey system can be calculated as follows:

(19) $${\vec{X}}_{\mathrm{ECEF}}={\vec{X}}_{\mathrm{INS}}^{\mathrm{ECEF}}+T_{\mathrm{INS}}^{\mathrm{ECEF}}\cdot \left(T_{L}^{\mathrm{INS}}\cdot \vec{s}+{\vec{X}}_{L}^{\mathrm{INS}}\right)$$

where ${\vec{X}}_{\mathrm{ECEF}}$ is the coordinates of the point on the ground in the ERF, ${\vec{X}}_{\mathrm{INS}}^{\mathrm{ECEF}}$ is the offset vector from the IRF to the ERF, $T_{\mathrm{INS}}^{\mathrm{ECEF}}$ is the rotation matrix from the IRF to the ERF, $T_{L}^{\mathrm{INS}}$ is the rotation matrix from the LRF to the IRF, $\vec{s}$ is the position of the laser footprint on the ground in the LRF, and ${\vec{X}}_{L}^{\mathrm{INS}}$ is the offset vector from the LRF to the IRF.

**Figure 9:**
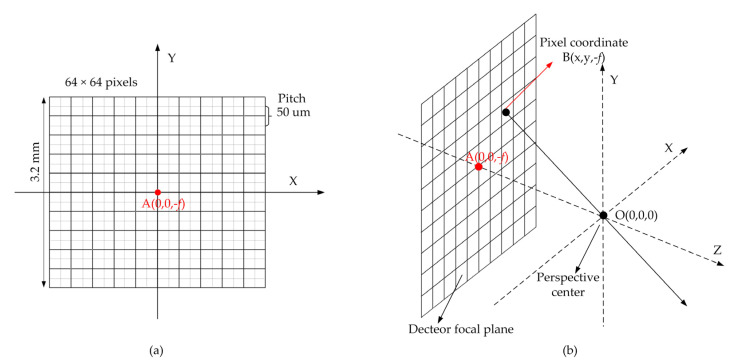
Diagram of the lidar coordinate system and ray unit vector: (**a**) the 2D view of the lidar coordinate system; (**b**) the 3D view of the lidar coordinate system.

For lidar employing a GMAPD array, the establishment of the lidar geometric model is different from the lidar system with a single detector. For our GML system, the laser was only used to illuminate the surface, and the detector shot a picture with distance, as shown in Figure 2. According to the reversibility of light theory, we assumed that the light emits from the pixel in the detector array. The light-emitting unit vector could be easily determined through a ray-tracing model. As described in [21], we defined the lidar’s coordinate system *O_xyz_*, as shown in Figure 9. The origin of the LiDAR coordinate system *O* is the perspective center of the lidar imaging system. The *O_z_*-axis is perpendicular to the detector focal plane and points to the ground along the optical axis of the imaging system. The axis *O_x_*-axis and *O_y_*-axis are parallel to the side of the array, respectively, according to the right-hand system.

As shown in Figure 9b, the focal length of the imaging system is *f*, the array center is denoted by coordinate A (0, 0, *−f*), and each pixel coordinate is denoted by B (x, y, *−f*). The value of x and y is dependent on the pixel pitch of the array and the pixel location in the array. Therefore, the ray unit vector can be calculated by:

(20) $${\vec{I}}_{0}=\frac{\vec{BO}}{\|\vec{BO}\|}$$

**Figure 10:**
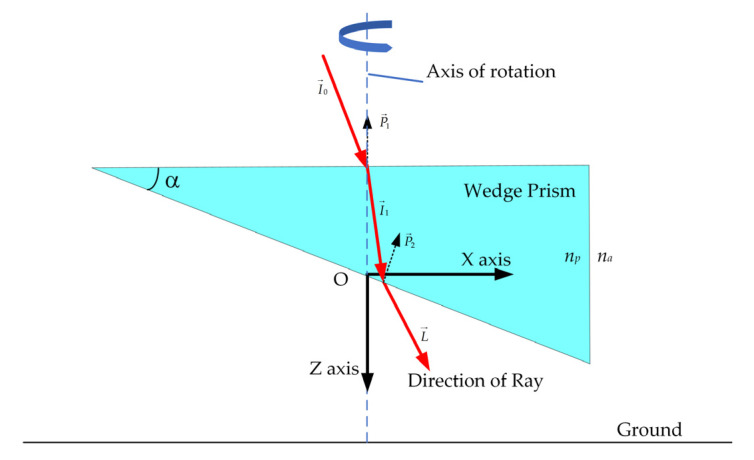
Diagram of the wedge prism frame and tracking the rays’ path in, through, and out of the rolling wedge prism.

When the ray is propagated to the scanner, the path of the ray through the wedge prism can be traced by Snell’s Law. The diagram of the prism-scanner assembly is shown in Figure 10. In this circumstance, we defined the scanner coordinate system as the same as the lidar coordinate system. The rotated angle given by the encoder was defined as zero. Therefore, the initial two surface normal vectors for the upper and lower facets of the wedge prism can be given by:

(21) $${\vec{P}}_{1}={\left[\begin{array}{ccc} 0 & 0 & 1 \end{array}\right]}^{T}$$

(22) $${\vec{P}}_{2}={\left[\begin{array}{ccc} \sin(\alpha ) & 0 & -\cos(\alpha ) \end{array}\right]}^{T}$$

where α is the slope of the wedge prism.

However, the normal vector of the upper face and the lower face of the wedge prism will be changed during the rotation of the scanner. Because the scanner rotates the prism about its *Z*-axis, the scanner rotation transform matrix can be written as:

(23) $$T_{z}=\left[\begin{array}{ccc} \cos(\theta_{s}) & -\sin(\theta_{s}) & 0\\ \sin(\theta_{s}) & \cos(\theta_{s}) & 0\\ 0 & 0 & 1 \end{array}\right]$$

where $\theta_{s}$ is the rotated instantaneous angular position of the wedge prism measured by the encoder.

Hence, the normal vectors for the upper and lower facets of the wedge prism after rotation can be determined by Equations (24) and (25):

(24) $${\vec{N}}_{1}=T_{z}\cdot {\vec{P}}_{1}$$

(25) $${\vec{N}}_{2}=T_{z}\cdot {\vec{P}}_{2}$$

Based on the vector version of Snell’s Law [26], the direction of the laser exiting prism $\vec{L}$ can be calculated by Equations (26) and (27):

(26) $${\vec{I}}_{1}=\frac{n_{a}}{n_{p}}{\vec{I}}_{0}+\left(-\frac{n_{a}}{n_{p}}\left({\vec{N}}_{1}\cdot {\vec{I}}_{0}\right)-\sqrt{1-{\left(\frac{n_{a}}{n_{p}}\right)}^{2}\left(1-{\left({\vec{N}}_{1}\cdot {\vec{I}}_{0}\right)}^{2}\right)}\right){\vec{N}}_{1}$$

(27) $$\vec{L}=\frac{n_{p}}{n_{a}}{\vec{I}}_{1}+\left(-\frac{n_{p}}{n_{a}}\left({\vec{N}}_{2}\cdot {\vec{I}}_{1}\right)-\sqrt{1-{\left(\frac{n_{p}}{n_{a}}\right)}^{2}\left(1-{\left({\vec{N}}_{2}\cdot {\vec{I}}_{1}\right)}^{2}\right)}\right){\vec{N}}_{2}$$

where $n_{a}$ is the refractive index of air and $n_{p}$ is the refractive index of prism.

The ranging information measured in the pixel is *r*, and the point position in the lidar coordinate system can be determined by Equation (28):

(28) $$\vec{s}=r\cdot \vec{L}$$

Finally, the point geo-referencing in the ECEF reference frame can be calculated from Equations (19)–(28).

## 4. Experiment Results and Discussion

The results were obtained by several initial flight tests conducted in Wuhu City and Qionghai City with an aircraft velocity of 220 km/h. The airborne experiment was conducted in Wuhu City from 11:50 a.m. to 03:00 p.m. (UTC+8) on 14 January 2021, to validate the performance of the airborne GML system at altitudes of 1 km and 3 km. Then, a marine and terrestrial mapping task were implemented in Qionghai City during broad daylight from 28 January to 02 February 2021. The flying altitudes were 0.35 km and 0.5 km.

The gathered raw data were delivered to the data-decompression and point cloud generation modules to calculate the point coordinates in the ECEF reference frame. Then, a noise-filtering process was executed to clean the background noise point in the point clouds. Finally, the 3D point clouds were used to produce digital surface and terrain models, relative reflectance imagery, and other applied products. Approximately 8 TB point cloud products were generated during our whole flight test, due to the GML’s high-density images.

### 4.1. Positional Accuracy

**Figure 11:**
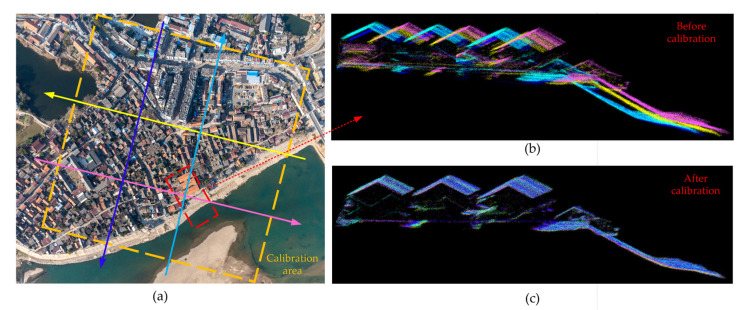
(**a**) The aerial camera image of Yijiang Town, Wuhu City. Four flight directions with different heading angles are marked by different colors. (**b**) Point cloud of three buildings and tilted riverbank before the calibration from an AGL of 1 km. (**c**) Point cloud of three buildings and tilted riverbank after the calibration from an AGL of 1 km.

**Figure 12:**
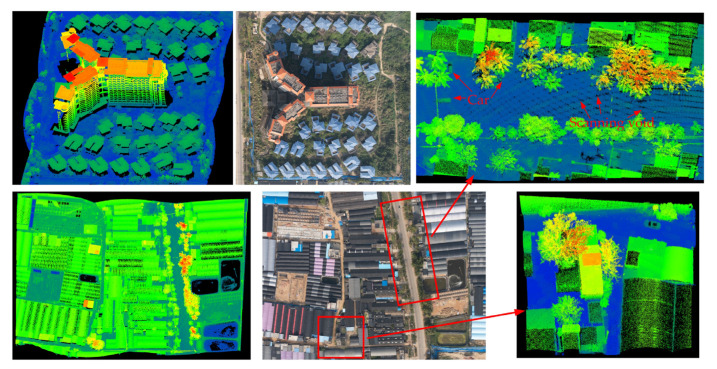
The GML system point cloud picture and digital color photograph of the aquaculture region in Huiwen Town, Qionghai City. The lidar images are colored according to the surface elevation (blue = low, red = high).

The calibration between lidar and INS sensors was conducted in Yijiang Town, Wuhu City. The calibration field was chosen over a group of surfaces with flat or tilted featuring as shown in Figure 11a. Ten calibration strips of GML data were collected, and these strips were perpendicular or parallel to each other. By selecting the planar features in the calibration area, the extrinsic parameters errors were corrected through the extrinsic self-calibration method [30]. The before and after calibration of the lidar image are shown in Figure 12b and Figure 12c, respectively. The point cloud quality was obviously improved.

According to the calculation method in [31], 20 tilted roofs and 20 flat surfaces were chosen to evaluate the relative positional accuracy. The standard deviation of the relative altimetric accuracy was 0.131 m, and the standard deviation of the relative planimetric accuracy was 0.152 m after the calibration. Therefore, the extrinsic parameter errors were eliminated significantly.

### 4.2. Sample Data

#### 4.2.1. Flight Altitude 0.35 km

**Figure 13:**
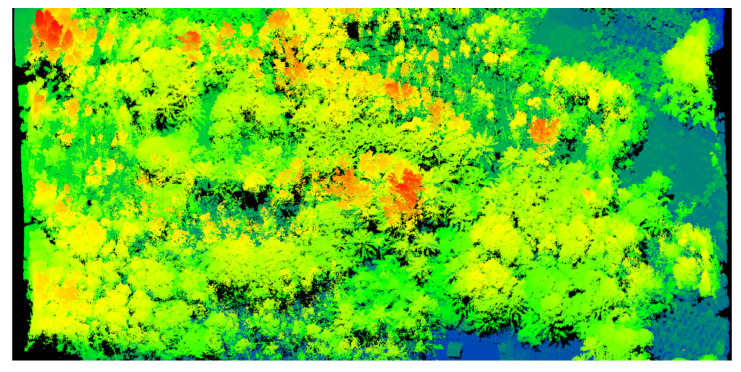
The GML image shows a 3D point cloud of a heavily foliated area. The canopy, tree trunk, and ground are presented clearly. The mean point density in this image is 1498.3 pts/m^2^.

Example images from airborne data collections over Huiwen Town, Qionghai City are shown in Figure 12 and Figure 13. These two figures were collected during a single pass over the surveying area at the flight altitude of 0.35 km. The buildings, coconut palms, cars, and power lines are distinctly presented in the lidar image. The mean measurement density of the lidar images was greater than 1400 points per square meter (pts/m^2^). The ponds in the lidar image are dark, reflecting back very little of the laser light at the near-infrared band. As shown in Figure 12, it is obvious that the scanning voids appeared in the adjacent forward swath. The rotational speed was set to 2000 RPM, which was lower than the optimal speed. Although the backward swath filled most scanning voids in the same area, the impact of a low rotational speed could not be canceled. Therefore, a higher rotational speed motor was required at low flight altitude.

#### 4.2.2. Flight Altitude 0.5 km

**Figure 14:**
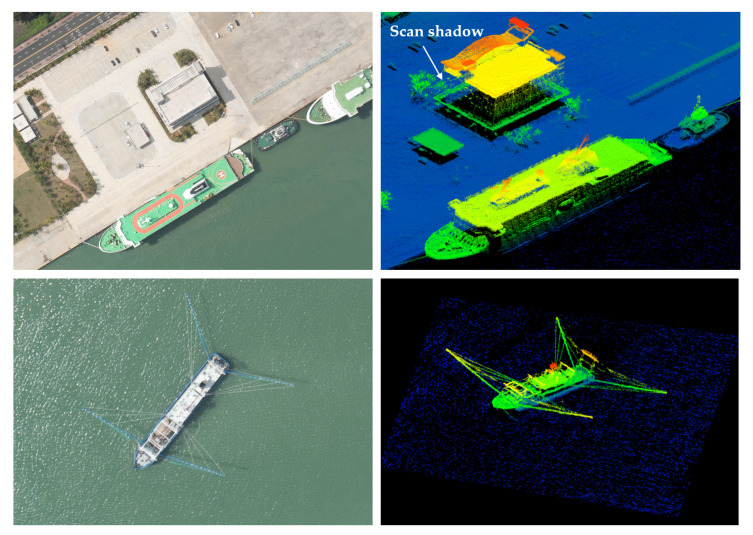
**Left:** The aerial picture of the passenger ship and trawl boat docked at Wenchang City and offshore ocean area, respectively. **Right:** The lidar image of the ships was acquired by a single pass at the altitude of 0.5 km and the mean point density is 598.8 pts/m^2^. The point cloud is colored according to its elevation. The dark blue in the lidar image is the ocean surface.

High-resolution 3D lidar images generated by the GML system over a passenger ship and trawl boat at the altitude of 0.5 km, are shown in the right of Figure 14. A similar view of the vessels captured by an aerial camera is displayed on the left. The feature details of the ships can be identified clearly in the high-density 3D lidar imagery. The scan shadows occurred in certain areas around the buildings when the data were acquired by a single pass. These scan shadows could be significantly reduced when flown with a 50 percent overlap. Four looks were provided by the scan pattern from four different directions, to reduce scan shadows [8]. In addition, the mean point density of the ocean surface reached 17.6 pts/m^2^, owing to the single-photon sensitivity of the Geiger-mode FPA detectors. The elevation of the ocean surface has potential application in marine monitoring and offers high-accuracy ocean surface ranging information to the linear-mode MWOL for range calibration.

#### 4.2.3. Flight Altitude 1 km

**Figure 15:**
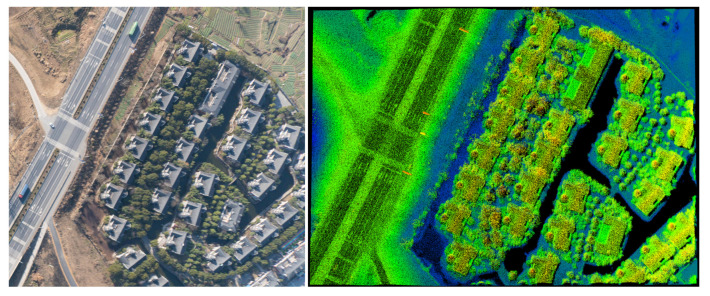
The visible-camera image and high-resolution elevation data across a villa complex in Yijiang Town. The color of the lidar image was set according to height. The dark areas are waterways.

Photograph and lidar images of a villa complex was collected by a single pass from 1 km, as shown in Figure 15. The visible-camera image and the lidar image were obtained at different times. Thus, the vehicles did not appear in the lidar image. The buildings, trees, crosswalks, and guideposts on the highway can be distinguished in the lidar image. The mean point density was 98.6 pts/m^2^ in this area. Although the GML could not obtain the targets’ reflected intensity information, the road markings could be identified due to the high reflectivity of the road markings. Therefore, the local point density of the GML image could be applied to obtain the relative reflectivity of the target for classification.

#### 4.2.4. Flight Altitude 3 km

**Figure 16:**
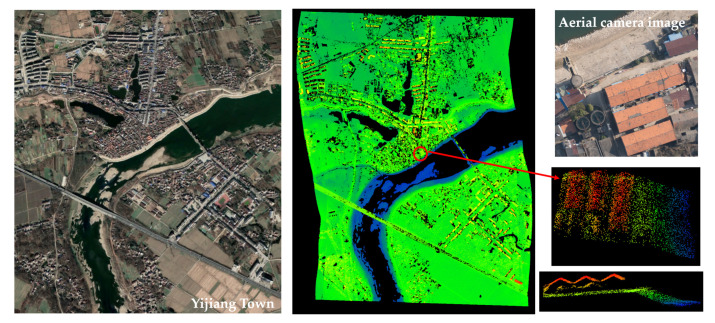
The satellite imagery (**left**) of Yijiang Town from Google Earth. The lidar image (**right**) was obtained on the same day as the satellite imagery, and the point cloud is colored to correspond to the change in elevation.

Figure 16 shows the lidar image across Yijiang Town from an AGL of 3 km. Aerial photos could not be obtained, as they were limited by the operating temperature of the camera. Therefore, the satellite imagery of Yijiang Town was used as a reference. The mean measurement density was 2.6 pts/m^2^ with an area collection rate of 366 km^2^/h.

### 4.3. System Optimization

By selecting 30 segments of point cloud data from each AGL flight dataset, the calculated mean point density of experiment and simulation results were obtained, as shown in Table 3. The point density of the airborne experiments agreed with the theoretical predictions. As shown in Table 2, a deviation is exhibited between the theoretical and experimental values, due to a low signal-to-noise ratio for detection at the altitude of 3 km. Therefore, the simulation model lidar could be used to predict the performance and design requirements of the GML system.

As the arrival times of the photons follow a Poisson process and the detector triggers just once for each laser pulse, the number of noise photons has an obvious influence on signal detection probabilities with the flight altitude raised. Generally, a smaller receiver FOV can significantly reduce the received noise photons. This led to scanning voids at a low flight altitude. In such a case, a higher rotational speed is required, which increases the scanner’s instability. An alternative solution to reducing solar background can be achieved through a narrower optical filter. Table 4 shows that the point density was significantly increased after replacing the 3 nm wide narrow-band optical filter with a 1 nm one. Moreover, the point cloud quality could support United States Geological Survey (USGS) Quality Level 1 data (8 pts/m^2^) at an altitude of 3 km. Therefore, appropriate system designs to reduce the number of background noise photons can significantly increase operating AGL and data quality, and reduce the requirement for noise filtering.

### 4.4. Discussion

To compare the performance of LML and GML, the RIEGL VQ-1560 II was chosen as the comparator. The RIEGL VQ-1560 II is a state-of-the-art commercial linear mode lidar [32]. Table 5 shows a performance comparison between the RIEGL VQ-1560 II and the GML system. A dual laser was used in RIEGL VQ-1560 II and the average laser power was 10 W for each [33]. For the RIEGL VQ-1560 II, when the laser power level was 12% and flight altitude was 750 m, the RIEGL VQ-1560 II and our GML had almost the same area coverage rates.

As shown in Table 5, although the laser power of the RIEGL VQ-1560 II was nine times higher than that of the laser power of the GML system, the point density of the GML was nearly twice as large as the point density of the RIEGL VQ-1560 II. Moreover, the main dimensions, weight, and power consumption of the RIEGL VQ-1560 II were much larger in contrast to the GML system. However, the range precision of the RIEGL VQ-1560 II was much better than that of the GML system. Future work is needed to improve the ranging accuracy/precision of the GML system. In addition, the vegetation penetration capability of GML is worse than that of LML, because each pixel in the GMAPD array has only one measurement opportunity per imaging cycle. Nevertheless, the vegetation penetration capability of GML could be promoted when the array detector enables multiple ToF measurements per APD per cycle [1].

Owing to the high sensitivity array detector, the GML system can acquire the high-density point cloud with huge area coverage rates. Recently, it has been used in military surveillance, disaster relief, floodplain mapping, and biomass measurement [18]. For the potential applications, the GML has a greater detection distance with an eye safety laser energy and it can be used in autonomous driving. In addition, the GML could be more portable and can be mounted on the unmanned aerial vehicle for civilian mapping.

## 5. Conclusions

In this study, we presented a simulation model of circular scanning airborne Geiger-mode FPA imaging lidar and developed a GML system. The system can operate day and night and the operating AGL is between 0.35 km and 3 km. We used an eye-safe low-power fiber laser at 1545 nm and a 64 × 64 pixels photon-counting detector array in the system. The RMS range precision of the flat buildings was 0.09 m. The proposed real-time data-compression algorithm reduced half of the raw data transmission rate from 156 MB/s to 78 MB/s. For the lidar employing a GMAPD array, we presented the lidar geometric model according to the ray-tracing model and the reversibility of light theory. Then, the point cloud generation module was applied to produce lidar images. In addition, we presented samples from recently collected high-resolution 3D data at different altitudes. The positional accuracy of the point cloud was evaluated after the calibration of lidar and INS sensors. The standard deviation of the relative altimetric accuracy was 0.131 m. The standard deviation of the relative planimetric accuracy was 0.152 m. The measured point density agreed with the theoretical predictions of the simulation model. The experiment results indicated that real-time data compression can improve the performance of the airborne GML system. Therefore, these results point the way toward the practical implementation of designing an airborne GML system and the data compression method. Future work will focus on the performance promotion of the system and the application of the GML system in mapping, high-resolution 3D imaging, foliage-penetration, and disaster relief.

## Figures and Tables

**Table 1:** Summary table of design properties for the GML system.

	Specification
Wavelength	1545 nm
Laser repetition rate	20 kHz
Laser pulse width (FWHM)	0.7 ns
Laser average power	260 mW
Beam divergence	5.5 mrad
Telescope diameter	75 mm
GMAPD array pixels	64 × 64
Pixel pitch	50 μm
Effective fill factor	0.6
Detector efficiency	0.2
Dark count (each pixel)	5 kHz
Instantaneous field of view	0.06 mrad
Timing resolution	1 ns
Time gate	4096 ns
Spectral filter (FWHM)	3 nm
Optical efficiency	0.5
Scan pattern	Circular
Scan angle	Fixed 31°
Flight altitudes	0.35–3 km
Weight	15 kg
Maximal power	150 W/28 VDC

**Table 2:** The simulation results of the GML system at different flight altitudes.

Flight Altitude (km)	Signal Photons	Noise Photons	Point Density (pts/m^2^)	Rotational Speed (RPM)	
				Minimum	Optimum
0.35	1.627	1.866	1366.8	2629	2686
0.5	0.797	1.866	645.2	1840	2247
1	0.199	1.866	107.6	920	1589
1.5	0.089	1.866	33.7	613	1298
2	0.050	1.866	14.5	460	1124
2.5	0.032	1.866	7.5	368	1005
3	0.022	1.866	4.3	307	917

**Table 3:** A comparison of experimental and simulation results in mean point density.

AGL	0.35 km	0.5 km	1 km	3 km
Simulation	1366.8 pts/m^2^	645.2 pts/m^2^	107.6 pts/m^2^	4.3 pts/m^2^
Experiment	1406.2 pts/m^2^	658.1 pts/m^2^	112.4 pts/m^2^	2.6 pts/m^2^

**Table 4:** The simulation results of the GML system by just reducing the optical filter bandwidth from 3 nm to 1 nm.

Flight Altitude (km)	Signal Photons	Noise Photons	Point Density (pts/m^2^)
0.35	1.627	0.636	3439.1
0.5	0.797	0.636	1646.2
1	0.199	0.636	270.7
2	0.050	0.636	36.4
3	0.022	0.636	10.9

**Table 5:** Performance comparison between the RIEGL VQ-1560 II and the GML system.

System	RIEGL VQ-1560 II [32]	Our GML
Laser wavelength	1064 nm	1545 nm
Laser beam divergence	0.25 mrad	5.5 mrad
Average laser power	2 × 1200 mW	260 mW
Laser repetition rate	2 × 2000 kHz	20 kHz
Measurements per second	2.66 million	81.92 million
Receiving elements	2 × 1	4096
Range precision	2 cm	9 cm
Flight altitude	750 m	1000 m
Scan FOV	58°	31°
Area coverage rates	128 km^2^/h	122 km^2^/h
Point density	60 pts/m^2^	112.4 pts/m^2^
Main dimensions	Ø 524 mm × 780 mm	235 mm × 355 mm × 298 mm
Weight	55 kg	15 kg
Maximum power consumption	550 W	150 W

## Data Availability

Not applicable.
